# Effects of combined inoculation of arbuscular mycorrhizal fungi and plant growth-promoting rhizosphere bacteria on seedling growth and rhizosphere microecology

**DOI:** 10.3389/fmicb.2024.1475485

**Published:** 2025-01-07

**Authors:** Wanli Zeng, Dan Xiang, Xuemei Li, Qian Gao, Yudong Chen, Kunmiao Wang, Yingying Qian, Luoping Wang, Jing Li, Qili Mi, Haitao Huang, Li Xu, Mingfang Zhao, Yingzhen Zhang, Haiying Xiang

**Affiliations:** ^1^Yunnan Academy of Tobacco Science, Kunming, China; ^2^College of Resources and Environment, Qingdao Agricultural University, Qingdao, China

**Keywords:** arbuscular mycorrhizal fungi, growth attributes, microecological mechanisms, plant growth-promoting rhizobacteria, illumina sequencing

## Abstract

The effects of rhizosphere microorganisms on plant growth and the associated mechanisms are a focus of current research, but the effects of exogenous combined inoculation with arbuscular mycorrhizal fungi (AMF) and plant growth-promoting rhizobacteria (PGPR) on seedling growth and the associated rhizosphere microecological mechanisms have been little reported. In this study, a greenhouse pot experiment was used to study the effects of single or double inoculation with AM fungi (*Funneliformis mosseae*) and two PGPR (*Bacillus* sp., *Pseudomonas* sp.) on the growth of tobacco seedlings, together with high-throughput sequencing technology to reveal associated rhizosphere microecological mechanisms. All inoculation treatments significantly increased the aboveground dry weight; root dry weight; seedling nitrogen, phosphorus, and potassium uptake; plant height; stem thickness; maximum leaf area; chlorophyll content; total root length, surface area, and volume; and average root diameter. The highest values for these indices were observed in the combined treatment of *F*. *mosseae* and *Pseudomonas* sp. SG29 (A_SG29). Furthermore, the A_SG29 treatment yielded the highest diversity indexes and largest percentages of significantly enriched bacterial taxa, and significantly promoted the colonization of AMF in tobacco roots and *Pseudomonas* in rhizosphere soil. Differential metabolic-pathway predictions using PICRUSt2 showed that the A_SG29 treatment significantly increased the metabolic pathway richness of tobacco rhizosphere microorganisms, and significantly up-regulated some metabolic pathways that may benefit plant growth. Co-inoculation with *F*. *mosseae* and *Pseudomonas* sp. SG29 promoted tobacco-seedling growth by significantly improving rhizosphere microbial communities' structure and function. In summary, the combined inoculation of AMF and SG29 promotes tobacco seedling growth, optimizes the rhizosphere microbial community's structure and function, and serves as a sustainable microbial co-cultivation method for tobacco seedling production.

## 1 Introduction

Arbuscular mycorrhizal fungi (AMFs) are beneficial fungi that form reciprocal symbiotic relationships with the root systems of ~90% of vascular plants worldwide, promoting mineral nutrient uptake, improving plant stress tolerance, and enhancing plant growth. Their strong underground mycelial networks enable host-to-host signaling and mitigate tolerance to stressful environments; these are essential factors in plant growth and development (Del-Saz et al., 2017; Wang et al., 2021). Mycorrhizae formation between AMFs and tobacco roots significantly increases the root volume, root area, total root length, and root vigor of tobacco plants, while improving root morphological structure and plant nutritional status (Jiang et al., 2017). Mycorrhizae also activate nutrient elements around the root system, improve plant nutrient utilization, enhance disease resistance, improve stress tolerance, improve the rhizosphere microenvironment, effectively alleviate stress damage, and increase biomass accumulation (Subhashini, 2016; Naheeda et al., 2021). Moreover, they promote the biosynthesis of aromatic substances in tobacco leaves and improve the balance of sugar, nicotine, and chlorine elements in roasted tobacco leaves; overall, these effects improve tobacco quality (Naheeda et al., 2021; Ravnskov et al., 2019).

Plant growth-promoting rhizobacteria (PGPRs) are rhizosphere bacteria that promote crop nutrient uptake, inhibit pathogenic microorganisms, alleviate abiotic stresses, and secrete phytohormones, which constitute important factors that promote plant growth and maintain soil ecological health (Ahmad et al., 2008; Lugtenberg and Kamilova, 2009; Gao et al., 2022). By fixing nitrogen (N), dissolving phosphorus (P), releasing potassium (K), secreting iron carriers, and producing plant hormones, PGPRs improve soil fertility and the stress resistance and nutrient statuses of tobacco plants, directly or indirectly promoting the growth of those plants (Subhashini, 2016; Liu et al., 2020; He et al., 2022; Shang et al., 2023; Zhang et al., 2024; Jian et al., 2024).

There is increasing evidence that co-inoculation with AMF or PGPR can significantly promote plant growth and yield. For example, double inoculation with AMF and PGPR synergistically promotes the growth of tomato, maize, cereal, pigeon pea, rice, wheat, and other plants (Mathimaran et al., 2020; Sagar et al., 2021; Ji et al., 2022; Baniyaghob et al., 2024). However, antagonistic or neutral effects between AMFs and PGPRs have also been reported (Hidri et al., 2019; Nathalie et al., 2020). These findings suggest that differences among inoculum species, plant species, environmental conditions, and rhizosphere microbial communities lead to distinct AMF–PGPR interactions (John et al., 2020; Cai et al., 2021), highlighting the need for further research. Thus far, few studies have explored the synergistic effects of AMFs and PGPRs on tobacco growth (Mesbah et al., 2021). Nursery seedling establishment is the optimal stage for inoculating plants with beneficial microorganisms; this approach ensures that such microorganisms come into direct contact with the plants' root systems for early establishment of symbiotic relationships (Anith et al., 2015; Angúlo-Castro et al., 2021). The rhizosphere microbiome is a microdomain environment that links the plant and soil; therefore, it also responds to PGPR or AMF inoculation (Chen et al., 2022). The synergistic effects of rhizosphere microbial communities were often neglected in previous studies investigating potential mechanisms related to the effects of AMFs and PGPRs on plant growth.

Therefore, in this study, we examined the effects of co-inoculation of exogenous PGPRs and AMFs on tobacco growth, as well as the mechanism underlying the rhizosphere microbial growth promotion effects. We conducted single or combined inoculations using two PGPRs (*Bacillus* sp. SG42 and *Pseudomonas* sp. SG29) and one AMF (*Funneliformis mosseae*) on tobacco seedling growth. We performed high-throughput sequencing analysis to compare rhizosphere soil bacterial communities among various inoculation treatment conditions. The objective of this study was to provide a theoretical basis and technical support for research and development focused on tobacco composite microbial seedling fertilizers.

## 2 Materials and methods

### 2.1 Materials

We used *F. mosseae* as the AMF inoculant in this study. Regarding the two PGPRs used as inoculants, *Pseudomonas* sp. SG29 (hereinafter, SG29) exhibits N fixation, P solubilization (organic and inorganic P solubilization indices = 3.43 and 1.93, respectively), K solubilization (K solubilization index = 2.07), indole-3-acetic acid (IAA) production, and acetyl-CoA carboxylase dehydrogenase production. The other PGPR used as an inoculant was *Bacillus* sp. SG42 (hereinafter, SG42), which exhibits N fixation and P solubilization (organic P solubilization index = 3.83). All tested strains were cultured in our laboratory. The AMF inoculum, consisting of spores and mycelium, was propagated in a medium that comprised a mixture of grass charcoal, peat, vermiculite, in which corn (*Zea mays*), clover (*Trifolium repens*), and sorghum (*Sorghum bicolor*) had been grown for 5.5 months to obtain mature spores. The inoculum density was 2,000 spores per gram of substrate. The preparation of PGPR bacterial suspension is as follows: The strains were inoculated on sterile basic LB liquid medium (35°C, 130 r·Min^−1^ constant temperature culture) for 12–16 h, centrifuged at 10,000 r·min^−1^ for 10 min. The bacteria were precipitated and diluted with sterile distilled water to OD600 value to a uniform quantitative concentration (1 × 10^8^ CFU/mL), and 10 ml of bacteria solution was added to each plant. The test plant was tobacco (*Nicotiana tabacum* L.).

The substrate comprised 60% grass carbon, 20% vermiculite, and 20% perlite (pH 6.11), with an alkali-hydrolyzed N content of 320 mg/kg, total N content of 6.10 g/kg, available P content of 210 mg/kg, available K content of 1,513 mg/kg, and organic matter content of 395 g/kg.

### 2.2 Experimental design

Six treatments were established: control without inoculation (CK), single inoculation with AMF, single inoculation with SG42, single inoculation with SG29, double inoculation with AMF and SG29 (hereinafter, A_SG29), and double inoculation with AMF and SG42 (hereinafter, A_SG42). Each AMF inoculated plant received 2.5 g of AMF through inoculation (5,000 spores per pot), and all uninoculated plants were treated with the same amount of inocula that had been autoclaved twice at 121°C for 120 min. Each treatment included 100 seedlings, which were grown in a climate chamber at 20–28°C and watered according to dryness and humidity. The plants were grown for 55 days from sowing to harvest.

### 2.3 Measurement indices and methods

#### 2.3.1 Plant biomass and nutrient content

After harvest, we randomly selected 20 tobacco seedlings for determination of agronomic traits, including plant height, stem diameter, maximum leaf length, and maximum leaf width, in accordance with the standards of the State Tobacco Monopoly Bureau of China (YC/T142-2010). Leaf area was calculated as leaf length × leaf width × 0.6345. Chlorophyll content was determined using a Soil Plant Analysis Development (SPAD) instrument. To obtain the dry mass of the aboveground parts and root system, the aboveground plant parts and root system of each seedling were divided, rinsed with tap or distilled water, and patted dry with absorbent paper; they were subsequently oven-dried at 105°C for 30 min and at 70°C until a constant weight was reached. Total N concentration was determined after plant digestion using the Kjeldahl method (Thilakarathna et al., 2019), total P concentration was determined using the vanadium–molybdenum yellow colorimetric method (Zhang et al., 2020), and total K concentration was measured using the flame photometric method (Zhang et al., 2020). Plant N, P, and K content was calculated by multiplying their respective concentrations by the dry weight.

#### 2.3.2 Determination of mycorrhizal colonization rates

At harvest, 10 tobacco seedlings were randomly selected, and root samples were randomly collected from the whole root system of each plant, cut into segments of ~1 cm, stained with trypan blue, and observed under a microscope, as previously described (Phillips and Hayman, 1970). The mycorrhizal colonization rate was calculated using Mycocalc (https://www2.dijon.inrae.fr/mychintec/Mycocalc-prg/download.html).

#### 2.3.3 Root scanning

For root measurements, 10 plants were randomly selected from each treatment. Each plant was removed from its pot; the root system was separated from the aboveground parts and slowly rinsed with running water while collecting any residual roots to ensure maximum root integrity. A scanner (Expression 11000XL, Epson, Nagano, Japan) was used to scan the roots; WinRHIZO software was used to calculate the total root length, total root surface area, total root volume, and root diameter.

#### 2.3.4 MiSeq sequencing

Three rhizosphere soil samples were randomly selected from each treatment for bacterial diversity analysis. Total DNA from the rhizosphere soil of each tobacco seedling was extracted using the FastDNA Spin Kit for Soil (MP Biomedicals, Santa Ana, CA, USA), in accordance with the manufacturer's instructions. DNA integrity was detected by 1% agarose gel electrophoresis; DNA concentration and purity were detected using a nucleic acid quantifier (NanoDrop 2000; Thermo Fisher Scientific, Waltham, MA, USA). The primers 338F (5′-ACTCCTACGGGGAGGCAGCA-3′) and 806R (5′-GGACTACHVGGGGTWTCTAAT-3′) were selected for polymerase chain reaction (PCR) amplification of the V3–V4 region of the 16S rRNA gene of the sample bacteria (Claesson et al., 2009).

Polymerase chain reaction products were detected using 1% agarose gel electrophoresis, purified in accordance with the instructions of the AxyPrepDNA Gel Recovery Kit (Axygen Biosciences, Union City, NJ, USA), and quantified by QuantiFluor-ST (Promega, Madison, WI, USA). Sequencing was performed on the NovaSeq 6000 PE250 platform (Illumina, San Diego, CA, USA) with support from Personalbio (Shanghai, China).

### 2.4 Data processing and analyses

QIIME2 software was used to call DADA2 algorithms for quality control, denoising, splicing, and chimera removal (Bolyen et al., 2019); a sequence similarity of 97% was used as the threshold for classification of operational taxonomic units (OTUs). Representative OTU sequences were used for bacterial community composition analysis, α- and β-diversity analyses, and bacterial colony function prediction. R software (R Core Team, Vienna, Austria) and other tools were used to perform principal coordinate analysis (PCoA) based on the Bray–Curtis distances of the samples, combined with the Adonis distances. Difference tests were conducted by combining the PCoA results with the Adonis analysis results. Linear discriminant analysis effect size (LEfSe) assessments were performed in R. The Personalbio Genes Cloud platform (https://www.genescloud.cn) was used for analyses of bacterial community composition. Figures were drawn using Circos software. Phylogenetic Investigation of Communities by Reconstruction of Unobserved States (PICRUSt2) software was used for comparisons of bacterial OTU abundance matrices with Kyoto Encyclopedia of Genes and Genomes (KEGG) database entries to obtain functional prediction information for each sample (Langille et al., 2013). The metagenomeSeq package in R was used to detect differential microbe abundances among samples, with a threshold of *P* < 0.05. KEGG Orthology (KO) IDs associated with metabolism were selected to draw a clustering heatmap in R.

Sequencing data were uploaded to the National Center for Biotechnology Information (NCBI) Sequence Read Archive database (accession nos. PRJNA1066034 and SAMN39477885–SAMN39477902).

For each indicator, means were compared using the analysis of variance method in DPS v7.5 software, followed by Tukey's test to detect significant pairwise differences, with a threshold of *P* < 0.05.

## 3 Results

### 3.1 Tobacco seedling growth and nutrient uptake

**Figure 1:**
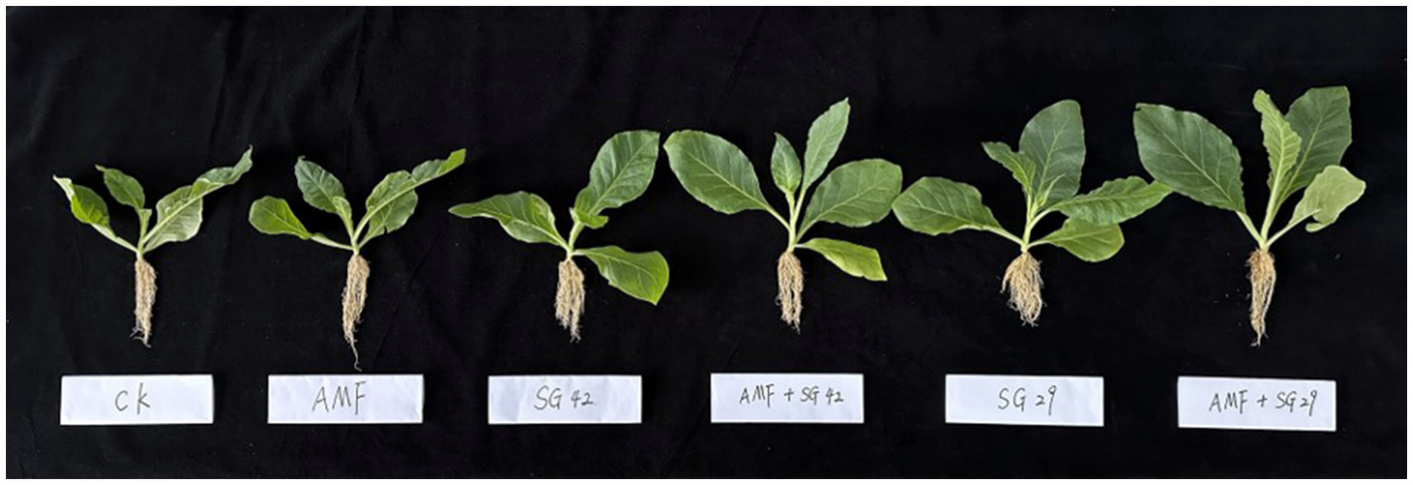
Tobacco seedling growth phenotypes under different treatments.

**Figure 2:**
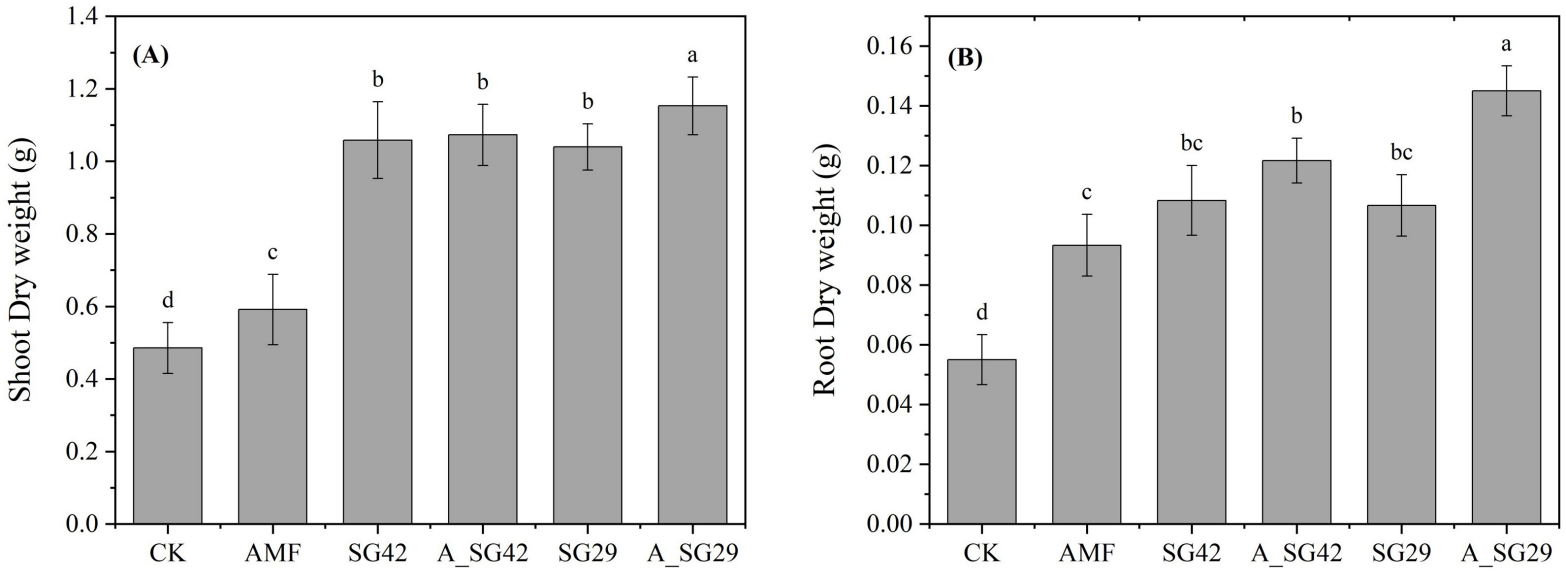
Shoot dry weight **(A)** and root dry weight **(B)** of tobacco seedlings in various arbuscular mycorrhizal fungus (AMF) and plant growth-promoting rhizobacterium (PGPR) treatments. Treatment codes are explained in Section 2.2. Different letters indicate significant differences according to Tukey's honestly significant difference test (*P* < 0.05).

Tobacco seedlings in all inoculated treatments exhibited significantly greater aboveground dry weight and root dry weight compared with CK (Figures 1, 2). Double inoculation treatments receiving A_SG29 had the highest aboveground dry weight and root dry weight; these were 135% and 150% higher, respectively, compared with CK and significantly higher than the corresponding weights in other treatments. The aboveground dry weight of the single-inoculation AMF treatment was only 21.91% higher than the weight of CK, whereas the aboveground dry weights of both single and combined PGPR inoculation treatments were at least 114.21% higher than the weight of CK; these results indicated that PGPR treatment had a better growth promotion effect than AMF treatment in terms of aboveground growth. Single inoculation with AMF led to a 69.70% increase in root dry weight compared with CK, which did not significantly differ from the effects of single inoculation with either PGPR strain but was significantly lower than the weight after double inoculation; these results suggested that AMFs have root promotion effects similar to PGPRs, and that the synergistic effects of AMF and PGPR treatments are beneficial to root growth.

Tobacco seedling nutrient content indices for each inoculation treatment are listed in Table 1. All inoculation treatments significantly increased seedling N, P, and K contents compared with CK; the highest increases (162.5%, 97.32%, and 76.43%, respectively) were observed under double inoculation with A_SG29. Notably, these uptake rates were significantly higher than the rates for all single-inoculation treatments and CK. No significant differences in seedling N, P, or K content were observed between single inoculation with PGPR and double inoculation with PGPR and AMF. All inoculation treatments significantly enhanced the uptake of nitrogen (N), phosphorus (P), and potassium (K) by seedlings, with the double inoculation treatment showing the most significant effects.

**Table 1:** Effects of different treatments on agronomic traits, root growth, and arbuscular mycorrhizal fungus (AMF) root colonization of tobacco seedlings.

	**CK**	**AMF**	**SG42**	**A _SG42**	**SG29**	**A _SG29**
Plant height (cm)	2.63 ± 0.47^d^	3.08 ± 0.64^d^	4.31 ± 0.63^c^	5.28 ± 0.84^b^	6.53 ± 0.83^a^	5.36 ± 0.63^b^
Steam diameter (mm)	5.55 ± 0.32^d^	6.13 ± 0.46^c^	7.56 ± 0.34^b^	7.72 ± 0.50^b^	7.66 ± 0.48^b^	8.19 ± 0.45^a^
Maximum leaf area (cm^2^)	90.17 ± 7.47^d^	100.75 ± 9.4^c^	125.01 ± 12.32^b^	129.63 ± 8.81^b^	129.43 ± 16.84^b^	144.99 ± 15.15^a^
SPAD value	29.43 ± 2.29^d^	32.96 ± 1.84^c^	39.30 ± 2.00^a^	35.34 ± 3.42^b^	38.57 ± 2.84^a^	39.40 ± 1.75^a^
Root length (cm)	1068.77 ± 41.11^c^	1467.09 ± 85.30^b^	1568.31 ± 73.25^b^	1556.35 ± 55.99^b^	1540.40 ± 47.83^b^	1899.22 ± 65.06^a^
Root area (cm^2^)	84.09 ± 5.13^c^	108.24 ± 5.94^b^	116.86 ± 9.77^b^	140.30 ± 9.16^a^	118.54 ± 5.33^b^	147.61 ± 7.71^a^
Root volume (cm^3^)	0.48 ± 0.06^c^	0.63 ± 0.05^b^	0.68 ± 0.07^b^	1.06 ± 0.03^a^	0.66 ± 0.03^b^	1.07 ± 0.08^a^
Average root diameter (mm)	0.22 ± 0.01^c^	0.25 ± 0.01^bc^	0.24 ± 0.02^c^	0.28 ± 0.02^a^	0.24 ± 0.02^c^	0.29 ± 0.02^ab^
AM root colonization (%)	–	20.13 ± 0.52^b^	–	29.21 ± 0.48^a^	–	30.83 ± 0.79^a^
N content (mg/plant)	1.76 ± 0.26^d^	2.44 ± 0.18^c^	3.61 ± 0.58^b^	4.13 ± 0.36^ab^	4.01 ± 0.43^ab^	4.62 ± 0.23^a^
P content (mg/plant)	4.47 ± 0.66^c^	6.46 ± 0.30^b^	7.15 ± 0.76^ab^	7.75 ± 0.92^a^	7.72 ± 0.57^a^	8.82 ± 0.91^a^
k content (mg/plant)	4.37 ± 0.45^c^	5.35 ± 0.37^b^	7.69 ± 0.48^a^	6.89 ± 0.54^a^	7.41 ± 0.25^a^	7.71 ± 0.68^a^

Treatment codes are explained in Section 2.2. Different letters on the bars indicate significant differences according to Tukey's HSD at *P* < 0.05.

### 3.2 Agronomic traits of tobacco seedlings

Inoculation with microbial agents significantly improved the agronomic traits of tobacco plants (Table 1). All inoculation treatments significantly increased the stem thickness, maximum leaf area, and chlorophyll content of tobacco seedlings compared with CK. Among all inoculation treatments, the greatest stem thickness, maximum leaf area, and chlorophyll content values were observed in double-inoculation treatments (AMF + SG29), with respective increases of 47.57%, 60.80%, and 33.88%; these values also were significantly greater than the values in all other treatments. All treatments (including single inoculation and mixed inoculation with PGPR) showed significantly better agronomic traits (such as plant height, stem diameter, maximum leaf area, and chlorophyll content) compared to the single inoculation with AMF treatment. This result indicated that PGPR has a more significant effect on improving agronomic traits of tobacco seedlings than AMF. In addition, the mixed inoculation of A_SG42 showed no significant difference in all indicators compared to the single inoculation of SG42, while the indicators of the mixed inoculation of A_SG29 were significantly higher than those of the single inoculation of SG29. This observation suggests that SG29 may exhibit a stronger synergistic interaction with AMF, thereby enhancing its effectiveness.

### 3.3 Root morphology of tobacco seedlings

**Figure 3:**
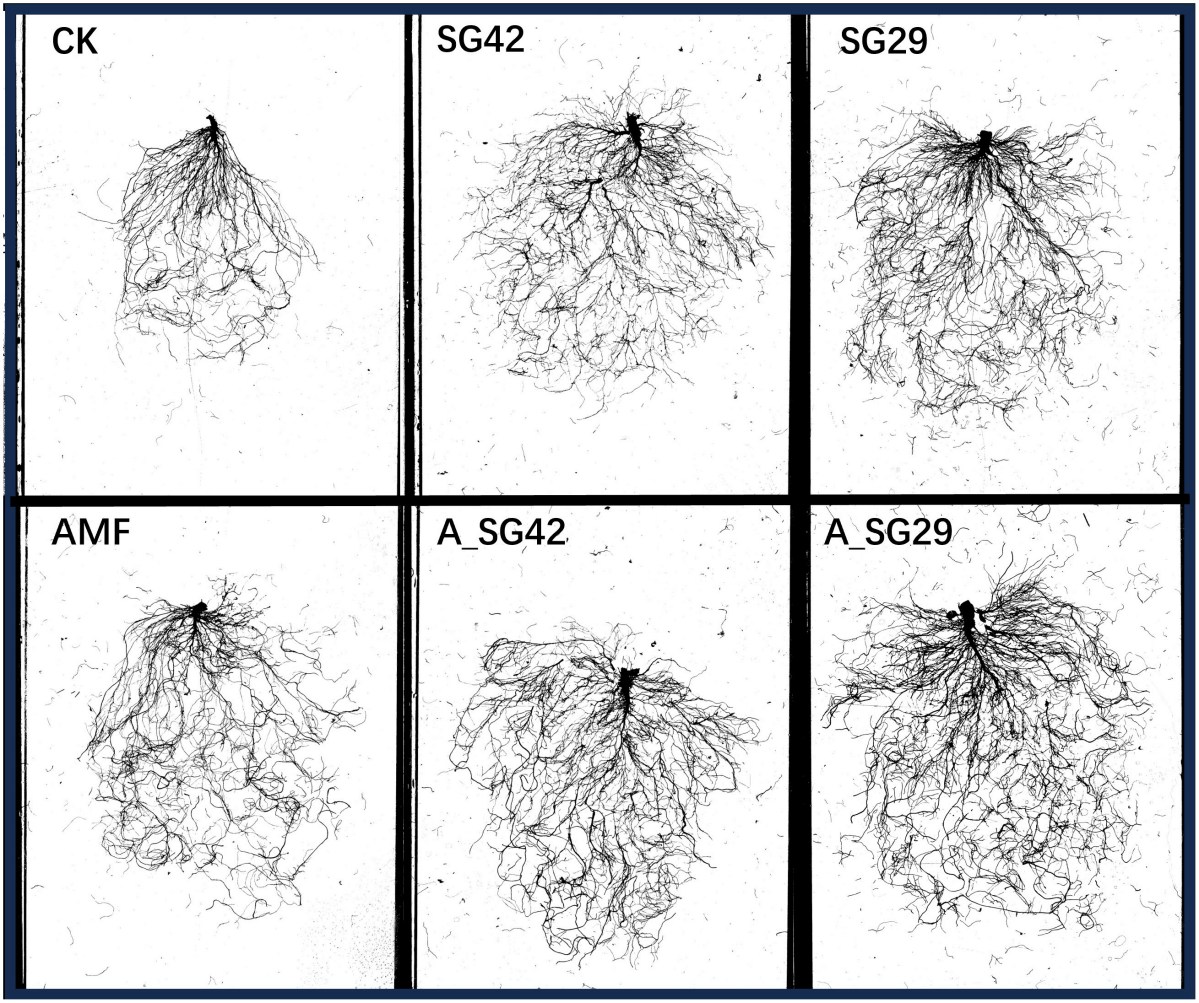
Scans of tobacco root systems developed under different treatments.

Seedling total root length, total surface area, total root volume, and average root diameter were significantly greater in all inoculation treatments than in CK (Table 1 and Figure 3). Treatment A_SG29 showed the greatest increases compared with CK, reaching 77.70%, 75.54%, 122.92%, and 31.82%, respectively; these increases were significantly greater than the increases in all other treatments. The dual inoculation treatments (A_SG29 and A_SG42) exhibited significantly higher indicators compared to any of the single inoculation treatments. In contrast, the three single-inoculated treatments did not demonstrate significant differences in their indices. This suggests that there was no notable difference between the individual effects of AMF and PGPR on root morphology; however, a significant synergistic effect was observed between these two factors.

### 3.4 AMF colonization of tobacco seedling roots

No mycorrhizal structures were detected in the root systems of tobacco seedlings among the three treatments not inoculated with AMF (Table 1). The mycorrhizal colonization rate of the treatment inoculated with AMF alone was 20.13%; the rates of combined inoculation treatments were significantly higher than the rates of single-inoculation treatments. The mycorrhizal colonization rates of the combined inoculation treatments (A_SG29 and A_SG42) increased by 45.11% and 53.15%, respectively, compared with single-AMF inoculation. These results indicated that combined inoculation with AMF and PGPR significantly enhanced mycorrhizal fungal colonization in the root systems of tobacco seedlings.

### 3.5 Bacterial α-diversity indices of tobacco seedling rhizosphere soil

The bacterial diversities of tobacco seedling rhizosphere soil samples were analyzed using multiple indices. The observed species index and Chao1 index were used to evaluate species richness; the Shannon and Simpson indices were used to evaluate bacterial community diversity. The observed species, Chao1, Shannon, and Simpson indices were highest in the A_SG29 treatment, indicating that bacterial community richness and diversity were greatest in this treatment (Table 2).

**Table 2:** Tobacco rhizosphere bacteria α-diversity indices under different treatments.

	**Observed_species**	**Chao1**	**Simpson**	**Shannon**
CK	2005.90 ± 101.65^b^	2033.25 ± 107.84^c^	0.9953 ± 0.0004^ab^	9.2322 ± 0.0619^c^
AMF	2115.33 ± 168.66^b^	2142.03 ± 173.57^bc^	0.9951 ± 0.0015^ab^	9.2597 ± 0.2372^bc^
SG42	2249.60 ± 47.66^ab^	2272.06 ± 40.82^abc^	0.9945 ± 0.0008^b^	9.3151 ± 0.0701^abc^
A _SG42	2319.13 ± 76.91^ab^	2373.76 ± 84.35^abc^	0.9963 ± 0.0000^ab^	9.5087 ± 0.0235^abc^
SG29	2501.27 ± 204.15^a^	2532.49 ± 201.00^ab^	0.9967 ± 0.0001^a^	9.6184 ± 0.0225^ab^
A _SG29	2526.27 ± 88.41^a^	2579.90 ± 81.18^a^	0.9970 ± 0.0002^a^	9.6691 ± 0.1142^a^

Diversity index values are means ± standard deviations. Different letters in the same column indicate significant differences (P < 0.05).

### 3.6 Bacterial β-diversity indices of tobacco seedling rhizosphere soil

**Figure 4:**
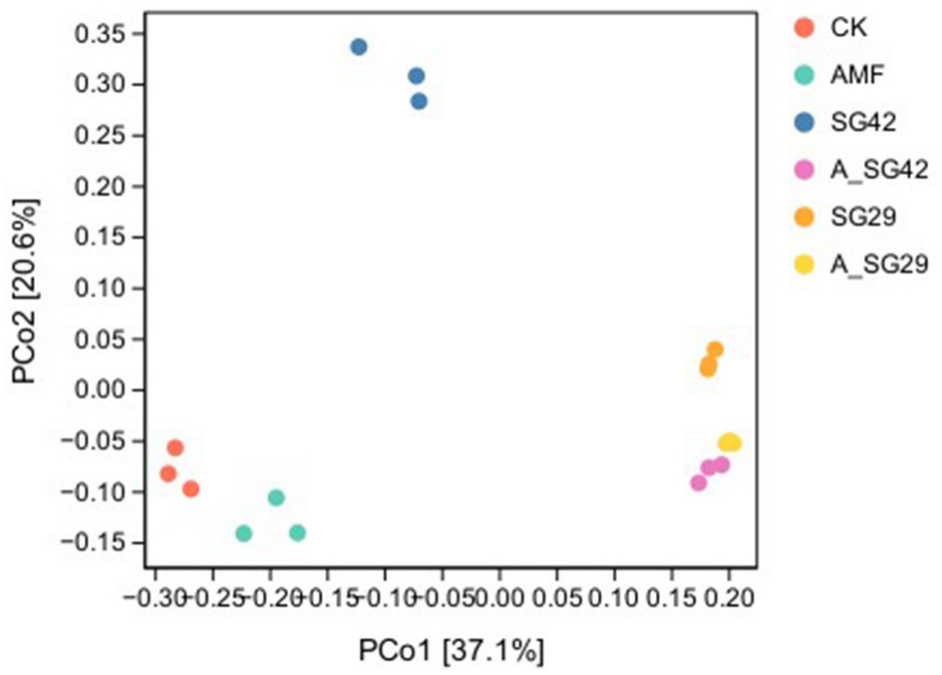
Bray–Curtis distance-based principal coordinates analysis (PCoA) of bacterial communities in tobacco rhizosphere soil under various treatments.

To clarify the extent of variation in bacterial community species composition among treatments, we performed PCoA to compare the β-diversities of rhizosphere soil bacterial communities among tobacco seedling treatments. The results showed that principal components 1 and 2 (PCo1 and PCo2) explained 37.1% and 20.6% of differences in community structure, respectively; collectively, these components explained 57.7% of such differences (Figure 4). The bacterial community compositions of the double-inoculated treatments (A_SG29 and A_SG42) showed a high degree of similarity, but there were significant differences between the single-inoculation SG29 and SG42 treatments. Furthermore, Adonis analysis revealed significant differences in the β-diversity of bacterial communities in the root systems of tobacco seedlings among treatments (R2 = 0.72213, *P* < 0.0001). This suggests that the inoculation treatment has significantly modified the community structure of rhizosphere soil microorganisms in tobacco seedlings.

### 3.7 Bacterial community compositions in tobacco seedling rhizosphere soil

**Figure 5:**
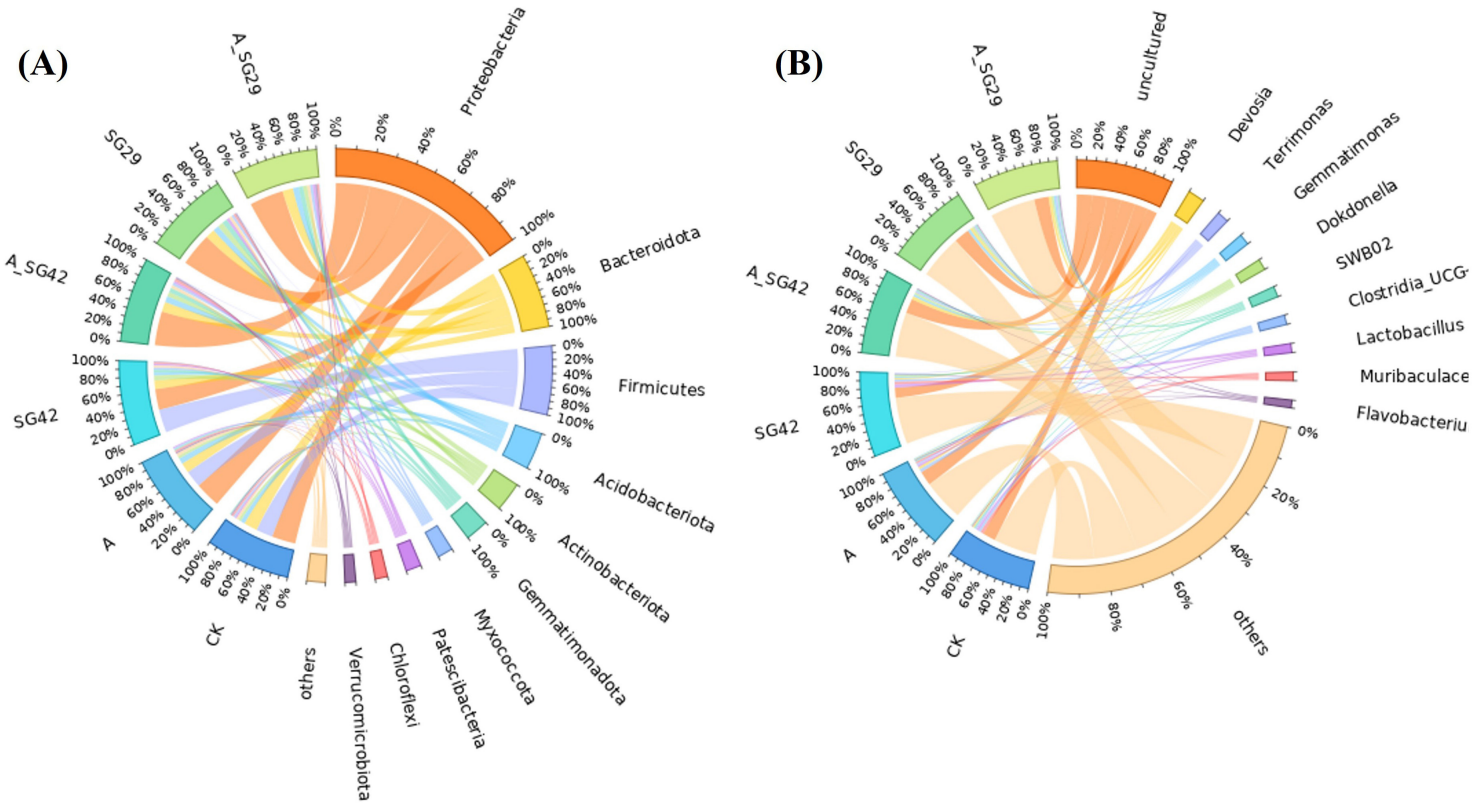
Distributions and abundances of the top 10 taxa in each sample at the phylum **(A)** and genus **(B)** levels under different treatments.

At the phylum level, bacterial community compositions in tobacco seedling rhizosphere soil were similar among treatments, although there were differences in relative abundance (Figure 5). The dominant phyla were Proteobacteria (40.05%), Bacteroidota (14.58%), Firmicutes (13.52%), Acidobacteriota (7.25%), Actinobacteriota (5.74%), Gemmatimonadota (4.55%), Myxococcota (3.06%), Patescibacteria (3.02%), Chloroflexi (2.46%), Verrucomicrobiota (2.12%), and others (3.66%). Proteobacteria abundances in the A_SG29, SG29, and A_SG42 treatments were 47.56%, 46.42%, and 47.64%, respectively; these were significantly higher than abundances in the CK (33.42%), AMF (36%), and SG42 (29.23%) treatments. In contrast, Firmicutes abundances were significantly higher in the SG42 (32.45%), AMF (23.36%), and CK (23.15%) treatments than in the A_SG29 (0.76%), SG29 (0.63%), and A_SG42 (0.76%) treatments. The dominant bacterial genera in tobacco seedling rhizosphere soil were Devosia (3.26%), Terrimonas (2.32%), Gemmatimonas (2.16%), Dokdonella (2.15%), SWB02 (2.11%), Clostridia (UCG-014; 1.85%), Lactobacillus (1.77%), Muribaculaceae (1.67%), Flavobacterium (1.51%), and BIrii41 (1.50%). Among these, Gemmatimonas and Dokdonella had significantly higher relative abundances in all treatments inoculated with PGPR (A_SG29, SG29, A_SG42, and SG42) than in the CK and AMF treatments. The above results indicate that the dual inoculation treatment significantly increased the relative abundance of dominant species in the rhizosphere soil of tobacco seedlings.

### 3.8 Differential bacterial taxa in tobacco seedling rhizosphere soil

**Figure 6:**
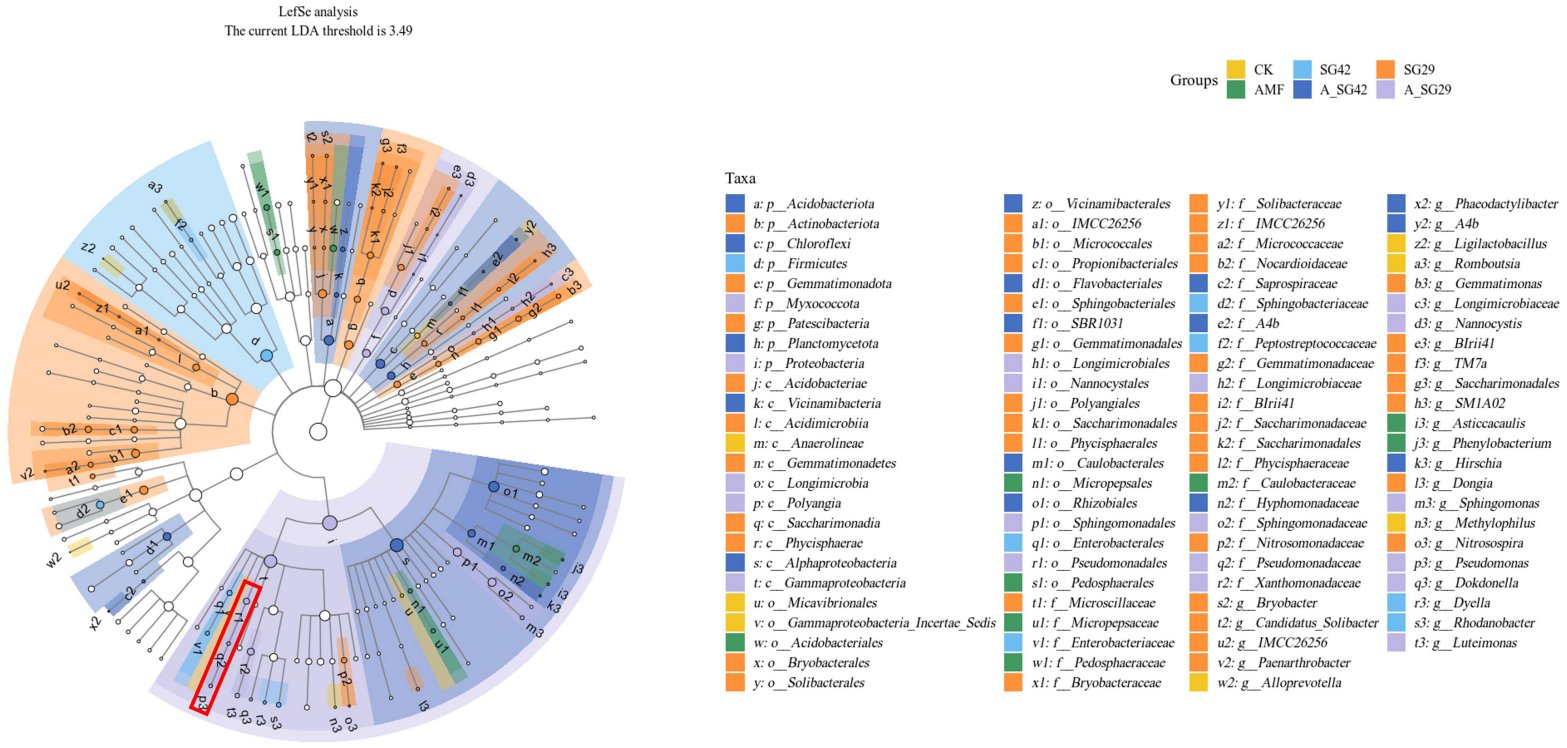
Cladograms indicating differences in taxa between treatments, generated through linear discriminant analysis (LDA) effect size (LEfSe) assessments using a significance threshold of 3.49. Red box indicates the group to which *Pseudomonas* sp. SG29 belongs.

Linear discriminant analysis effect size assessments were conducted to identify significantly different bacterial populations in tobacco seedling rhizosphere soil among treatments. Branching evolutionary diagrams revealed the presence of 100 significantly different bacterial taxa among the six groups. Of these taxa, 41, 19, and 16 were enriched in treatments SG29, A_SG29, and A_SG42, respectively; these values comprised significantly more taxa than were detected in the SG42 (9), AMF (8), and CK (7) treatments. This result suggested that treatments SG29, A_SG29, and A_SG42 experienced more pronounced changes in microbial community structure than the remaining treatments. At the gate level, the main taxon enriched by single inoculation with SG42 was Firmicutes, whereas the taxa most enriched by single inoculation with SG29 were Actinobacteriota, Gemmatimonadota, and Patescibacteria; taxa enriched by double inoculation of A_SG42 were Acidobacteriota, Chloroflexi, and Planctomycetota. Taxa enriched in the double-inoculation A_SG29 treatment included Proteobacteria and Myxococcota, whereas the CK and AMF treatments did not show significantly enriched taxa at the gate level (Figure 6). At the gate level for Proteobacteria, taxa enriched by treatments A_SG29 and A_SG42 were clearly differentiated: A_SG29 was enriched in Gammaproteobacteria, whereas A_SG42 was enriched in Alphaproteobacteria. The data presented in Figure 6 indicate a significant enrichment of the taxon associated with strain SG29 (Pseudomonas sp.) in the A_SG29 treatment group. Furthermore, as shown in Supplementary Figure 1, the relative abundance of “Pseudomonas” at the genus level was markedly higher in samples treated with SG29 compared to other treatment groups. This effect was particularly pronounced in the treatment group where AMF were co-inoculated with SG29, resulting in the highest observed relative abundance of “Pseudomonas.”. Similarly, the relative abundance of “Bacillus”—the genus associated with strain SG42—was significantly elevated in samples treated with SG42 compared to the control group (CK) and the group inoculated with AMF alone. Notably, the co-inoculation of AMF and SG42 yielded the maximum relative abundance of “Bacillus.”

### 3.9 Predictive analysis of microbial metabolic functions

**Figure 7:**
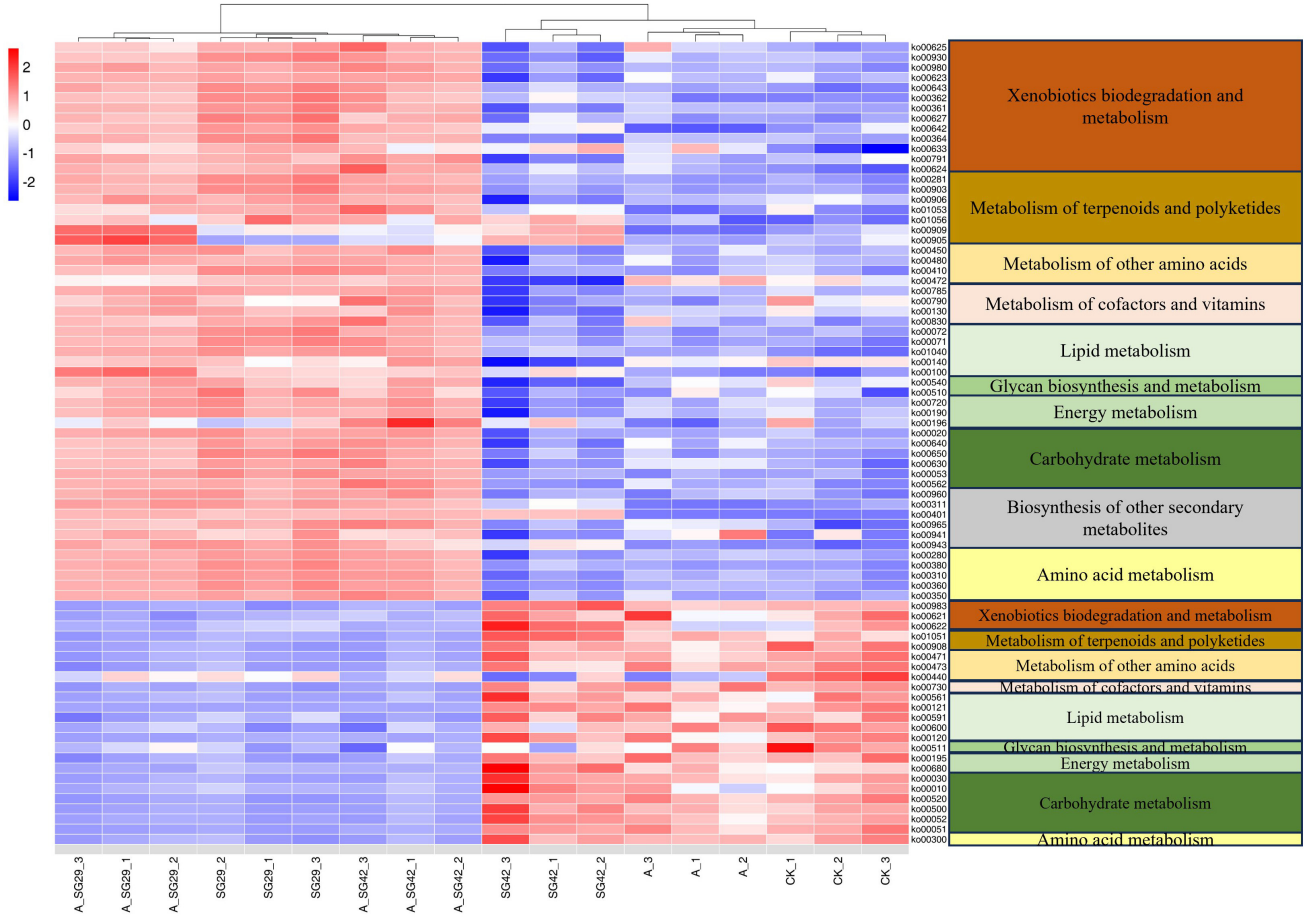
**(Left)** Abundance heatmap of Kyoto Encyclopedia of Genes and Genomes (KEGG) Orthology (KO) metabolic pathways in different treatments. **(Right)** Secondary metabolism pathways corresponding to KO numbers.

The metabolic functions of bacterial 16S rRNA gene sequences were predicted using PICRUSt based on KEGG database annotation. The abundance data for existing metabolic pathways were analyzed; differences in metabolic function among treatments were examined through pairwise comparisons of sample groups using the metagenomeSeq package in R. Metabolism-associated KOs were selected to create a clustering heatmap in R (Figure 7). The level 1–3 metabolic pathways corresponding to each KO number are listed in Supplementary Table 1. The six treatments were clustered into two classes: A_SG29, SG29, and A_SG42 were in one class, whereas SG42, CK, and AMF were in the other class. In total, 79 differential metabolic pathways were identified for the six treatments (P < 0.05). Compared with CK, 55 metabolic pathways in 10 classes of level 2 metabolic pathways were upregulated in treatments A_SG29, SG29, and A_SG42; 24 metabolic pathways in nine classes of level 2 metabolic pathways were downregulated in these three treatments. Additionally, seven terpenoid and polyketide metabolism pathways were upregulated and two such pathways were downregulated in treatments A_SG29, SG29, and A_SG42. All six secondary metabolite biosynthesis pathways were upregulated, including the flavonoid (ko00941) and isoflavonoid (ko00943) biosynthesis pathways. Furthermore, 13 xenobiotic biodegradation and metabolism pathways involved in the degradation of various organic substances were upregulated, whereas only three such pathways were downregulated. These results indicated that more functional genes in treatments A_SG29, SG29, and A_SG42 could utilize different carbon sources, and microbial metabolism was more active. Finally, the tryptophan metabolism pathway (ko00380) was significantly upregulated in the A_SG29 and A_SG42 treatments.

## 4 Discussion

Mycorrhizal colonization rates are important indicators that can be used to evaluate whether AMFs have established a symbiotic relationship with plants (Terlizzi et al., 2022). Higher mycorrhizal colonization rates are generally presumed to indicate more pronounced improvements in plant growth and stress tolerance (Zhang et al., 2018; Xiang et al., 2023). Previous studies have shown that combined inoculation with PGPR and AMF significantly promotes AMF colonization of plant root systems and increases AMF colonization rates (Barea et al., 2005; Pivato et al., 2009; Kim et al., 2010; Kumar et al., 2012). For example, *Pseudomonas fluorescens* sp. C7R12 promoted the colonization of *Glomus mosseae* sp. BEG12 in an alfalfa (*Medicago truncatula*) root system (Pivato et al., 2009). In another study, seven *P. fluorescens* soil isolates with PGPR traits were isolated; all seven isolates enhanced the colonization of sorghum root systems by *Glomus fasciculatum* and *Glomus aggregatum* (Kumar et al., 2012). However, there have been some conflicting reports. In one study, combined inoculation with a Bacillus PGPR and an AMF did not significantly affect AMF colonization rates in a goatgrass root system compared with single inoculation involving AMF (Yu et al., 2022). In contrast, another study revealed that peas (*Pisum sativum*) inoculated with both *Bacillus* sp. and *G. mosseae* showed significantly lower root mycorrhizal colonization rates than peas inoculated with *G. mosseae* alone; antagonism was detected between *G. mosseae* and *Bacillus* sp. (Bethlenfalvay et al., 1997). These findings suggest that there are large differences in effects among AMFs and PGPRs, as well as differences among host plants. Further studies are required to elucidate the effects of various combinations of specific microorganisms and host plants, enabling the selection of optimal combinations for crop growth and yield improvement. In the present study, two combined inoculation treatments (A_SG29 and A_SG42) increased the mycorrhizal colonization of tobacco roots by 53.15% and 45.11%, respectively, compared with single inoculation involving an AMF. These results indicated that combined inoculation with AMFs and PGPRs could significantly increase mycorrhizal fungal colonization in the root systems of tobacco seedlings. Both PGPR bacteria, *Pseudomonas* sp. SG29 and *Bacillus* sp. SG42, showed clear synergistic effects with AMFs. Predictive analysis of metabolic functions showed that the flavonoid and isoflavonoid biosynthesis pathways were significantly upregulated in rhizosphere soil sampled from both treatments that had been inoculated with mixtures of PGPRs and an AMF. Previous studies demonstrated that inoculation with PGPRs could promote flavonoid accumulation in basil and lettuce plants (Jung and Kim, 2020; Dasgan et al., 2022). Flavonoids can serve as signaling molecules during mycorrhizal formation and exhibit significant positive correlations with AMF colonization of plant roots (Smith and Read, 2008; Al-Ghamdi and Jais, 2013); such correlations likely represent an important mechanism by which both SG29 and SG42 increase AMF colonization rates in tobacco root systems.

AMFs and PGPRs are important components of the soil microbiota; single inoculation and co-inoculation treatments involving these microorganisms have demonstrated significant growth-promoting effects in plants (Mathimaran et al., 2020; Sagar et al., 2021; Cai et al., 2021; Ji et al., 2022; Baniyaghob et al., 2024). Similarly, our results showed that aboveground dry weight; root dry weight; plant height; stem diameter; maximum leaf area; chlorophyll content; total root length; total root surface area; total root volume; average root diameter; and the N, P, and K uptakes of tobacco seedlings were significantly increased in all inoculation treatments compared with CK. Thus, the AMF and both PGPRs showed clear growth promotion effects on tobacco seedlings; both PGPRs exhibited stronger effects, perhaps because these two strains actively promote nutrient uptake through IAA production and increases in acetyl-CoA carboxylase deaminase activity, ferric support, N fixation rates, P solubilization, and other functions. Chlorophyll synthesis and photosynthesis are enhanced through the promotion of nutrient uptake by plants (Siddiqui, 2006; Ahmad et al., 2008), as well as the absorption and transport of nutrients in leaves; these changes have additional enhancing effects on plant growth. Additionally, nearly all indices were higher in the combined inoculation treatments than in their corresponding single-inoculation treatments, the highest index values were observed in the combined *F. mosseae* + *Pseudomonas* sp. SG29 treatment, suggesting that although synergism between the AMF and either PGPR achieved beneficial effects, this effect was stronger between the AMF and SG29. Numerous studies have shown that *Pseudomonas* species are companion bacteria to AMFs (Hildebrandt et al., 2002; Roesti et al., 2005; Toljander et al., 2006), and some studies have revealed their synergistic effects (Kumar et al., 2012; Mathimaran et al., 2020; Chen et al., 2023). Although *Bacillus* is also a common semi-companion of AMF root systems, interactions substantially differ among microbial strains (Cai et al., 2021); some studies have demonstrated neutral or antagonistic interactions between *Bacillus* species and AMFs (Bethlenfalvay et al., 1997; Yu et al., 2022). These findings suggest that synergistic effects between *Pseudomonas* spp. and AMFs are stronger than synergistic effects involving *Bacillus* spp. Under our experimental conditions, inoculation with *Pseudomonas* sp. SG29 + *F. mosseae* had a significantly greater synergistic effect on tobacco growth promotion than co-inoculation with the *Bacillus* strain. This result is consistent with the findings of a recent study, in which double inoculation with a *Pseudomonas* strain and AMF had a significantly greater effect on rice biomass and plant N, P, and K uptakes compared with double inoculation involving a *Bacillus* strain and AMF (Chen et al., 2023).

The diversities and stabilities of rhizosphere microbial communities play important roles in plant growth and ecosystem sustainability (Brussaard et al., 2007). Greater microbial community structural complexity presumably contributes to soil ecosystem maintenance (Kennedy and Smith, 1995). In the present study, rhizosphere bacterial species richness and diversity indices exhibited different degrees of enhancement in seedlings across inoculation treatments; the highest α-diversity indices were detected in the double-inoculation SG29 + AMF treatment, followed by the A_SG42 treatment. β-diversity analysis also showed significant differences in microbial community structure among inoculation treatments. Specifically, LEfSe assessments revealed that the A_SG29 and A_SG42 treatments enriched more bacterial taxa than all other treatments, indicating that double inoculation with AMF and PGPR (particularly SG29 + AMF) significantly improved rhizosphere microbial community structure, which can stabilize the microbial ecosystem and enhance plant stress resistance (Deng et al., 2002).

The addition of bacteria to the plant rhizosphere can alter the rhizosphere microbial community (Thokchom et al., 2017). The present study showed that the inoculation with both SG29 and SG42 significantly enhanced the abundance of their respective taxa in the rhizosphere soil of tobacco seedings. These findings suggest that the two PGPR strains may achieve more effective colonization in the plant rhizosphere. Furthermore, co-inoculation appears to further augment the colonization capacity of PGPR. Despite these encouraging results, the lack of specific markers for the strains and the inherent methodological differences between high-throughput sequencing and plate culture techniques hinder a definitive confirmation of successful colonization. Nevertheless, it is clear that inoculation with these PGPR strains significantly increased the species abundance of their associated taxa. Additionally, LEfSe assessment demonstrated significant enrichment of taxa including *Pseudomonas* sp. SG29 in the double-inoculation A_SG29 treatment, implying that co-inoculation with AMF promoted its colonization in the tobacco seedling rhizosphere. This result is consistent with previous findings that AMF mycelial expansion and secretions in soil promoted root perimeter colonization by soil bacteria (Artursson et al., 2006). The A_SG29 treatment primarily enriched the taxon *Proteobacteria*, which has been identified as a key component of the “soil core microbiota” (Sun et al., 2024; Du et al., 2023). Members of this phylum possess the ability to catabolize both simple and complex carbohydrates, as well as aromatic compounds, and they harbor genes involved in critical biogeochemical processes, including carbon, nitrogen, phosphorus, and sulfur cycling within the maize rhizosphere (Li et al., 2014). Furthermore, a metaproteomics study demonstrated that *Proteobacteria*, along with *Actinobacteria*, accounted for the majority of bacterial proteins in the rice rhizosphere, underscoring their functional significance in rhizosphere ecosystems (Wang et al., 2010). Given its pivotal role in maintaining soil microecological health, *Proteobacteria* represents an essential component of the rhizosphere microbiome. The increased enrichment of this taxon in the A_SG29 treatment likely contributed to enhanced rhizosphere microbial functional diversity, highlighting the potential benefits of combined inoculation strategies. Differential metabolic pathway prediction by PICRUSt2 demonstrated that significantly more metabolic pathways were upregulated (55) than downregulated (24) in treatments A_SG29, SG29, and A_SG42 compared with CK, suggesting that these metabolic pathways are enriched in tobacco rhizosphere microorganisms exposed to these treatments. Among the xenobiotic biodegradation and metabolism pathways, 13 third-order pathways representing the degradation of various organic substances were upregulated, whereas only three such metabolic pathways were downregulated. The upregulated metabolic pathways were related to the degradation of toluene, styrene, benzoate, chlorocyclohexane, chlorobenzene, aminobenzoate, ethylbenzene, fluorobenzoate, nitrotoluene atrazine, and polycyclic aromatic hydrocarbon; such toxic organic substances are difficult to degrade in soil. This result indicates that the functional gene expression levels in microorganisms that use different carbon sources were increased in the A_SG29, SG29, and A_SG42 treatments; their metabolic functions were more active. However, these treatments also improved the toxicity resistance effects of the rhizosphere microbiome. Additionally, the A_SG29, SG29, and A_SG42 treatments upregulated seven terpenoid and polyketide metabolism pathways, while only downregulating two such pathways. Terpenoid metabolism is closely related to disease and stress resistance in plants (Li et al., 2017); therefore, this finding indirectly indicated that combined inoculation of AMF and PGPR and single inoculation of SG29 improved the disease and stress resistance capacities of tobacco seedlings. Furthermore, the tryptophan metabolism pathway (ko00380) was significantly upregulated in both double-inoculation treatments (A_SG29 and A_SG42). IAA is a metabolite of tryptophan; 82% of promastigotes (i.e., PGPRs) in rhizosphere soils utilize tryptophan or its intermediates to synthesize substances regulating plant growth and development (e.g., IAA, cytokinins, and gibberellins) and to stimulate the overgrowth of plant lateral roots and root hairs, thereby increasing the uptake of minerals, nutrients, and water necessary for plant growth (Gray and Smith, 2005; Tabassum et al., 2017; Fadiji et al., 2021). Significant upregulation of the tryptophan metabolism pathway may also be an important mechanism by which combined inoculation promotes tobacco growth. Because PICRUSt analysis only provides preliminary predictions of the functions of rhizosphere bacteria, further metabolomic studies are warranted.

## 5 Conclusions

This study demonstrated that all inoculation treatments significantly enhanced various growth parameters and nutrient uptake in tobacco seedlings. Specifically, the treatments increased aboveground dry weight, root dry weight, nitrogen (N), phosphorus (P), and potassium (K) uptake, plant height, stem thickness, maximum leaf area, chlorophyll content, total root length, total root surface area, total root volume, and mean root diameter. Among them, the double inoculation A_SG29 treatment showed the best results in all indicators. This indicated that both AMF fungi and both PGPR bacteria had significant growth promotion effects on tobacco seedlings, while the co-inoculation of *F. mosseae* and *Pseudomonas* sp. SG29 showed optimal synergistic effects in promoting the growth and improving the agronomic traits of tobacco seedlings. Furthermore, the high-throughput sequencing results indicated that the A_SG29 treatment yielded the highest diversity indexes and largest percentages of significantly enriched bacterial taxa. Further analysis of metabolic pathways using PICRUSt2 predicted that the A_SG29 treatment significantly increased the metabolic pathway richness of tobacco rhizosphere microorganisms, and significantly up-regulated some metabolic pathways that may benefit plant growth. Overall, our results indicated that co-inoculation with AMF and PGPR, particularly the co-inoculation of *F. mosseae* and *Pseudomonas* sp. SG29, promoted tobacco seedling growth by significantly improving rhizosphere microbial community structure and function. However, to fully understand the synergistic effects of the AMF–PGPR–tobacco triad, in-depth histological studies are required.

## Data Availability

The original contributions presented in the study are included in the article/Supplementary material, further inquiries can be directed to the corresponding author.
