# Supplementary material for: Effects of combined inoculation of arbuscular mycorrhizal fungi and plant growth-promoting rhizosphere bacteria on seedling growth and rhizosphere microecology
Source: Front Microbiol. 2025 Jan 7;15:1475485. doi: 10.3389/fmicb.2024.1475485 (PMC11758927; doi:10.3389/fmicb.2024.1475485)
Supplement: Supplementary file 1 [file Data_Sheet_1.docx]

**Supplementary Material**

Table S1 The level1,2,3 metabolic pathways of KEGG

| KO number | Level 1 | Level 2 | Level 3 |
| --- | --- | --- | --- |
| ko00625 | Metabolism | Xenobiotics biodegradation and metabolism | Chloroalkane and chloroalkene degradation |
| ko00930 | Metabolism | Xenobiotics biodegradation and metabolism | Caprolactam degradation |
| ko00980 | Metabolism | Xenobiotics biodegradation and metabolism | Metabolism of xenobiotics by cytochrome P450 |
| ko00623 | Metabolism | Xenobiotics biodegradation and metabolism | Toluene degradation |
| ko00643 | Metabolism | Xenobiotics biodegradation and metabolism | Styrene degradation |
| ko00362 | Metabolism | Xenobiotics biodegradation and metabolism | Benzoate degradation |
| ko00361 | Metabolism | Xenobiotics biodegradation and metabolism | Chlorocyclohexane and chlorobenzene degradation |
| ko00627 | Metabolism | Xenobiotics biodegradation and metabolism | Aminobenzoate degradation |
| ko00642 | Metabolism | Xenobiotics biodegradation and metabolism | Ethylbenzene degradation |
| ko00364 | Metabolism | Xenobiotics biodegradation and metabolism | Fluorobenzoate degradation |
| ko00633 | Metabolism | Xenobiotics biodegradation and metabolism | Nitrotoluene degradation |
| ko00791 | Metabolism | Xenobiotics biodegradation and metabolism | Atrazine degradation |
| ko00624 | Metabolism | Xenobiotics biodegradation and metabolism | Polycyclic aromatic hydrocarbon degradation |
| ko00281 | Metabolism | Metabolism of terpenoids and polyketides | Geraniol degradation |
| ko00903 | Metabolism | Metabolism of terpenoids and polyketides | Limonene and pinene degradation |
| ko00906 | Metabolism | Metabolism of terpenoids and polyketides | Carotenoid biosynthesis |
| ko01053 | Metabolism | Metabolism of terpenoids and polyketides | Biosynthesis of siderophore group nonribosomal peptides |
| ko01056 | Metabolism | Metabolism of terpenoids and polyketides | Biosynthesis of type II polyketide backbone |
| ko00909 | Metabolism | Metabolism of terpenoids and polyketides | Sesquiterpenoid and triterpenoid biosynthesis |
| ko00905 | Metabolism | Metabolism of terpenoids and polyketides | Brassinosteroid biosynthesis |
| ko00450 | Metabolism | Metabolism of other amino acids | Selenocompound metabolism |
| ko00480 | Metabolism | Metabolism of other amino acids | Glutathione metabolism |
| ko00410 | Metabolism | Metabolism of other amino acids | beta-Alanine metabolism |
| ko00472 | Metabolism | Metabolism of other amino acids | D-Arginine and D-ornithine metabolism |
| ko00785 | Metabolism | Metabolism of cofactors and vitamins | Lipoic acid metabolism |
| ko00790 | Metabolism | Metabolism of cofactors and vitamins | Folate biosynthesis |
| ko00130 | Metabolism | Metabolism of cofactors and vitamins | Ubiquinone and other terpenoid-quinone biosynthesis |
| ko00830 | Metabolism | Metabolism of cofactors and vitamins | Retinol metabolism |
| ko00072 | Metabolism | Lipid metabolism | Synthesis and degradation of ketone bodies |
| ko00071 | Metabolism | Lipid metabolism | Fatty acid degradation |
| ko01040 | Metabolism | Lipid metabolism | Biosynthesis of unsaturated fatty acids |
| ko00140 | Metabolism | Lipid metabolism | Steroid hormone biosynthesis |
| ko00100 | Metabolism | Lipid metabolism | Steroid biosynthesis |
| ko00540 | Metabolism | Glycan biosynthesis and metabolism | Lipopolysaccharide biosynthesis |
| ko00510 | Metabolism | Glycan biosynthesis and metabolism | N-Glycan biosynthesis |
| ko00720 | Metabolism | Energy metabolism | Carbon fixation pathways in prokaryotes |
| ko00190 | Metabolism | Energy metabolism | Oxidative phosphorylation |
| ko00196 | Metabolism | Energy metabolism | Photosynthesis - antenna proteins |
| KO number | Level 1 | Level 2 | Level 3 |
| ko00020 | Metabolism | Carbohydrate metabolism | Citrate cycle (TCA cycle) |
| ko00640 | Metabolism | Carbohydrate metabolism | Propanoate metabolism |
| ko00650 | Metabolism | Carbohydrate metabolism | Butanoate metabolism |
| ko00630 | Metabolism | Carbohydrate metabolism | Glyoxylate and dicarboxylate metabolism |
| ko00053 | Metabolism | Carbohydrate metabolism | Ascorbate and aldarate metabolism |
| ko00562 | Metabolism | Carbohydrate metabolism | Inositol phosphate metabolism |
| ko00960 | Metabolism | Biosynthesis of other secondary metabolites | Tropane, piperidine and pyridine alkaloid biosynthesis |
| ko00311 | Metabolism | Biosynthesis of other secondary metabolites | Penicillin and cephalosporin biosynthesis |
| ko00401 | Metabolism | Biosynthesis of other secondary metabolites | Novobiocin biosynthesis |
| ko00965 | Metabolism | Biosynthesis of other secondary metabolites | Betalain biosynthesis |
| ko00941 | Metabolism | Biosynthesis of other secondary metabolites | Flavonoid biosynthesis |
| ko00943 | Metabolism | Biosynthesis of other secondary metabolites | Isoflavonoid biosynthesis |
| ko00280 | Metabolism | Amino acid metabolism | Valine, leucine and isoleucine degradation |
| ko00380 | Metabolism | Amino acid metabolism | Tryptophan metabolism |
| ko00310 | Metabolism | Amino acid metabolism | Lysine degradation |
| ko00360 | Metabolism | Amino acid metabolism | Phenylalanine metabolism |
| ko00350 | Metabolism | Amino acid metabolism | Tyrosine metabolism |
| ko00983 | Metabolism | Xenobiotics biodegradation and metabolism | Drug metabolism - other enzymes |
| ko00621 | Metabolism | Xenobiotics biodegradation and metabolism | Dioxin degradation |
| ko00622 | Metabolism | Xenobiotics biodegradation and metabolism | Xylene degradation |
| ko01051 | Metabolism | Metabolism of terpenoids and polyketides | Biosynthesis of ansamycins |
| ko00908 | Metabolism | Metabolism of terpenoids and polyketides | Zeatin biosynthesis |
| ko00471 | Metabolism | Metabolism of other amino acids | D-Glutamine and D-glutamate metabolism |
| ko00473 | Metabolism | Metabolism of other amino acids | D-Alanine metabolism |
| ko00440 | Metabolism | Metabolism of other amino acids | Phosphonate and phosphinate metabolism |
| ko00730 | Metabolism | Metabolism of cofactors and vitamins | Thiamine metabolism |
| ko00561 | Metabolism | Lipid metabolism | Glycerolipid metabolism |
| ko00121 | Metabolism | Lipid metabolism | Secondary bile acid biosynthesis |
| ko00591 | Metabolism | Lipid metabolism | Linoleic acid metabolism |
| ko00600 | Metabolism | Lipid metabolism | Sphingolipid metabolism |
| ko00120 | Metabolism | Lipid metabolism | Primary bile acid biosynthesis |
| ko00511 | Metabolism | Glycan biosynthesis and metabolism | Other glycan degradation |
| ko00195 | Metabolism | Energy metabolism | Photosynthesis |
| ko00680 | Metabolism | Energy metabolism | Methane metabolism |
| ko00030 | Metabolism | Carbohydrate metabolism | Pentose phosphate pathway |
| ko00010 | Metabolism | Carbohydrate metabolism | Glycolysis / Gluconeogenesis |
| ko00520 | Metabolism | Carbohydrate metabolism | Amino sugar and nucleotide sugar metabolism |
| ko00500 | Metabolism | Carbohydrate metabolism | Starch and sucrose metabolism |
| ko00052 | Metabolism | Carbohydrate metabolism | Galactose metabolism |
| ko00051 | Metabolism | Carbohydrate metabolism | Fructose and mannose metabolism |
| ko00300 | Metabolism | Amino acid metabolism | Lysine biosynthesis |

Figure S1 Relative abundances of (a) *Bacillus*, to which SG42 belongs, and (b) *Pseudomonas*, to which SG29 also belongs, under different treatments.
